# Frailty and comorbidity burden in Atrial Fibrillation

**DOI:** 10.3389/fpubh.2023.1134453

**Published:** 2023-03-09

**Authors:** Francesco Salis, Antonella Palimodde, Giorgia Demelas, Maria Ilaria Scionis, Antonella Mandas

**Affiliations:** ^1^Department of Medical Sciences and Public Health, University of Cagliari, Cagliari, Italy; ^2^University Hospital “Azienda Ospedaliero-Universitaria” of Cagliari, Cagliari, Italy

**Keywords:** antiplatelet drugs, Atrial Fibrillation (AF), comorbidities, Comprehensive Geriatric Assessment, frailty

## Abstract

**Background:**

With the aging of the population, the characterization of frailty and comorbidity burden is increasingly taking on particular importance. The aims of the present study are to analyze such conditions in a population affected by Atrial Fibrillation (AF), matching it with a population without AF, and to recognize potential independent factors associated with such common cardiovascular disease.

**Methods:**

This study included subjects consecutively evaluated over 5 years at the Geriatric Outpatient Service, University Hospital of Monserrato, Cagliari, Italy. A sum of 1981 subjects met the inclusion criteria. The AF-group was made up of 330 people, and another 330 people were randomly selected to made up the non-AF-group. The sample was subjected to Comprehensive Geriatric Assessment (CGA).

**Results:**

In our sample, severe comorbidity burden (*p* = 0.01) and frailty status (*p* = 0.04) were significantly more common in patients with AF than without AF, independently on gender and age. Furthermore, the 5-years follow-up demonstrated that survival probability was significantly higher in AF-group (*p* = 0.03). The multivariate analysis (AUC: 0.808) showed that the presence of AF was independently positively associated with a history of coronary heart disease (OR: 2.12) and cerebrovascular disease (OR: 1.64), with the assumption of Beta Blockers (OR: 3.39), and with the number of drugs taken (OR: 1.12), and negatively associated with the assumption of antiplatelets (OR: 0.09).

**Conclusions:**

Elderly people with AF are frailer, have more severe comorbidities, and take more drugs, in particular beta blockers, than people without AF, who conversely have a higher survival probability. Furthermore, it is necessary to pay attention to antiplatelets, especially in AF-group, in order to avoid dangerous under- or over-prescriptions.

## Background

Global population is progressively aging (1, 2), and one of the roles that geriatric medicine has is to provide quality care for all these people (3). In fact, even if aging can be slowed down by lifestyle, it cannot be stopped (4, 5). A noble aim could be to early intercept mild deficits, and Comprehensive Geriatric Assessment (CGA) represent the specialistic tool which has the objective of achieving that (3), finding out how much support an elderly person needs for day-to-day living and helping to diagnose any health conditions they may have, by enquiring cognitive impairment, mood deflection, functional and nutritional status, other than comorbidities and quality of life (6–9).

Nowadays, in particular, it is used to assess “frailty”, a common medical word, whose interpretation is yet not univocal (10–12). It continues the literature continues to discuss “phenotypes” rather than “definitions”: the phenotypic model proposed by Fried et al. (13, 14) characterize frailty as a clinical syndrome with 3 or more criteria among weight loss, exhaustion, reduced grip strength, reduced walking speed and physical activity. Anyway, it is known that a categorization of pre-frail and frail people is necessary to stratify different needs for intervention (15). The concept of frailty is led to the concept of multimorbidity, which does not have to be considered as a “long list of illnesses”, but rather an indicator of burden (16), mortality (17), reduced quality of life (18) for elderly people. Anyways, multimorbidity is indeed associated with the most common geriatric syndromes, such as cognitive impairment and sarcopenia, and age-related pathologies (19–21). Among them, in cardiovascular medicine, one of the most represented in elderly is Atrial Fibrillation (AF). This common condition is an arrythmia which can be due to a number of factors including genetics, but also aging and lifestyle (22). It is associated with higher risk of hospitalization and higher mortality in elderly (23, 24).

## Methods

### Aim of the study

The primary aim of this study is to compare the frailty status and the comorbidity burden with the presence/absence of AF in a population of subjects aged 65 years or older, and to verify their impact on total mortality.

The secondary aim of this study is to consider which CGA domains, comorbidities and drugs are independently associated with AF.

### Design of the study

This observational cross-sectional study included subjects consecutively evaluated at the Geriatric Outpatient Service of the University Hospital of Monserrato, Cagliari, Italy, over a 5-years period.

### Inclusion criteria

Age ≥ 65 years; having been subjected to CGA.

### Exclusion criteria

Age < 65 years; age ≥ 65 years with acute conditions that contraindicated the CGA's execution; informed consent not provided.

One thousand nine hundred and eighty-one subjects met the inclusion criteria.

Non-valvular AF was present in 330 subjects (AF group): we performed a propensity score model to randomly match them with 330 non-AF controls (non-AF group) based on gender and age (see Section Statistical analysis).

We obtained a final sample of 660 subjects, who were followed-up for a 5-years period.

### Assessment

The enrolled subjects were evaluated with:

- Mini-Mental State Examination (MMSE) (25, 26) for cognitive assessment
- Geriatric Depression Scale (GDS) (27) for mood's assessment
- Basic Activities of Daily Living (ADL), Instrumental Activities of Daily Living (IADL) (28), Physical Performance Test (PPT) (29), and Performance Oriented Mobility Assessment (POMA) (30), for functional autonomy and physical performances' assessment
- Mini Nutritional Assessment (MNA) (31) for nutritional assessment
- Charlson Comorbidity Index (CCI) (32) for comorbidity burden's assessment
- FRAIL scale (14) for the categorization of the frailty level.

The abovementioned tests were administered by trained geriatricians in outpatient setting.

### Statistical analysis

Variables were expressed as means and standard deviations (SDs) or in percentages (%), were appropriate. In order to randomize cases and controls we used the propensity score: we firstly identified AF as “classification variable”, then, given a set of covariates, namely age and gender, we performed the test, obtaining neglectable 95% C.I. Kolmogorov-Smirnov test was used to check the distribution of quantitative data. Student's *t*-test test was used to compare continuous variables; chi-squared test (χ^2^) was used to compare qualitative variables. Correlations between variables were expressed using Pearson's rho (r). Multivariate analysis was performed with a logistic regression—stepwise (*p*-values > 0.1 were excluded by the model): its results were expressed as Odds Ratios (ORs) and confidence intervals (C.I.). Kaplan-Meier curves were designed in order to estimate the survival probability: in particular, mean survival times, expressed as Areas Under the survival Curves (AUC) from 0 to 5 years, were reported with their 95% C.I. The comparison of survival curves between the two groups was studied with the Logrank test, and expressed as χ^2^ and C.I., while the differences in time of occurring event were expressed as Hazard Ratios (HRs).

The results are reported indicating *p*-values in reference to 95% C.I.

MedCalc software (Version 19.5, Ostend, Belgium) was used for the statistical analysis.

## Results

**Figure 1:**
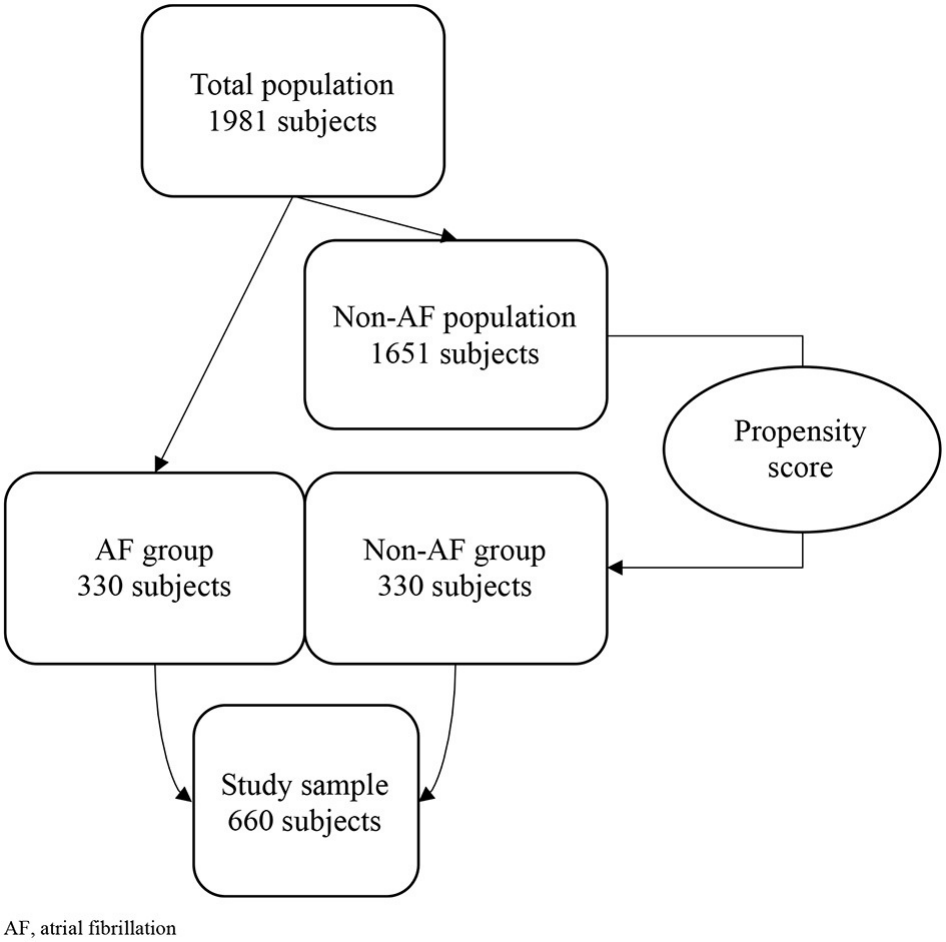
Study population. AF, atrial fibrillation.

The study included 1981 people aged 65 years or more. The propensity score model brought a final sample of 660 subjects, divided in two groups (AF and non-AF), of whom 414 women (62.7%), with average age of 81.2 years (SD: 6.5) (Figure 1).

Table 1 summarizes the scores achieved by the two groups in every CGA tests, and the most common co-morbidities and drugs taken.

**Table 1:** Characteristics of AF and non-AF group.

**Variable**	**AF (n. 330)**		**Non-AF (n. 330)**		***p*** **-value**	
**CGA**	**Mean**	**SD**	**Mean**	**SD**	***t*** **-test**	**K-S**
MMSE	20.2	6.7	19.8	6.7	0.4432	**< 0.0001**
GDS	8.0	3.9	7.8	3.9	0.5263	**< 0.0001**
ADL	64.6	24.7	62.4	24.5	0.2814	**< 0.0001**
IADL	2.5	2.4	2.2	2.1	0.0804	**< 0.0001**
PTT	9.8	5.8	9.0	5.1	0.0985	**< 0.0001**
POMA	13.2	6.7	12.7	6.6	0.3413	**< 0.0001**
MNA	19.2	4.6	19.1	4.5	0.7354	**< 0.0001**
BMI	27.9	5.3	26.9	5.1	0.1306	**0.0002**
CCI	6.6	2.1	6.2	2.1	**0.0222**	**< 0.0001**
FRAIL	2.9	1.4	2.6	1.4	**0.0109**	**< 0.0001**
Drugs taken (n.)	8.3	3.2	7.2	3.9	**0.0002**	**< 0.0001**
**Co-morbidities**	**%**		**%**		χ^2^	
Coronary heart disease	22.1		14.5		**0.0009**	
Cerebrovascular disease	35.8		33.0		0.1198	
Hypertension	84.8		79.4		0.2232	
COPD	29.4		18.5		**0.0001**	
Type 2 diabetes mellitus	28.2		25.5		0.4294	
**Drugs taken**	**%**		**%**		χ^2^	
Antiplatelet	24.8		56.4		**< 0.0001**	
VKA	45.5		2.4		**< 0.0001**	
DOAC	26.7		1.2		**< 0.0001**	
Diuretic	63.6		51.5		**0.0003**	
Beta blocker	48.8		23.0		**< 0.0001**	
Calcium channel blocker	25.2		23.9		0.8566	
CEI	33.6		30.0		0.4037	
ARB	31.5		34.5		0.1366	
Statin	33.6		33.3		0.9343	
PPI	56.4		48.2		**0.0239**	

AF, Atrial Fibrillation; CGA, Comprehensive Geriatric Assessment; SD, Standard Deviation; K-S, Kolmogorov-Smirnov test; MMSE, Mini Mental State Examination; GDS, Geriatric Depression Scale; ADL, Activities of Daily Living; IADL, Instrumental Activities of Daily Living; PPT, Physical Performance Test; POMA, Performance Oriented Mobility Assessment; MNA, Mini Nutritional Assessment; BMI, Body Mass Index; CCI, Charlson Comorbidity Index; COPD, Chronic Obstructive Pulmonary Disease; VKA, Vitamin K Antagonist; DOAC, Direct Oral Anticoagulant; CEI, angiotensin-Converting Enzyme Inhibitor; ARB, Angiotensin II Receptor Blockers; PPI, Proton-Pump Inhibitor.

Bold values indicate statistically significant.

We found that history of coronary heart disease (22.1 vs. 14.5%, *p* = 0.0009) and Chronic Obstructive Pulmonary Disease (COPD) (29.4 vs. 18.5%, *p* = 0.0001) were significantly more common in AF-group, as well as the assumption of Vitamin K Antagonists (VKA) (45.5 vs. 2.4%, *p* < 0.0001), Direct Oral Anti-Coagulants (DOAC) (26.7 vs. 1.2%, *p* < 0.0001), Beta Blockers (48.8 vs. 23%, *p* < 0.0001), and Proton-Pump Inhibitors (PPI) (56.4 vs. 48.2%, *p* = 0.0239), while antiplatelet drugs were more commonly taken by non-AF group (24.8 vs. 56.4%, *p* < 0.0001, of whom, respectively, 15 and 58% in secondary prevention). About CGA, CCI (6.6 vs. 6.2, *p* = 0.0222), FRAIL scores (2.9 vs. 2.6, *p* = 0.0109), and number of drugs taken (8.3 vs. 7.2, *p* = 0.0002) were higher in AF than in non-AF group.

**Figure 2:**
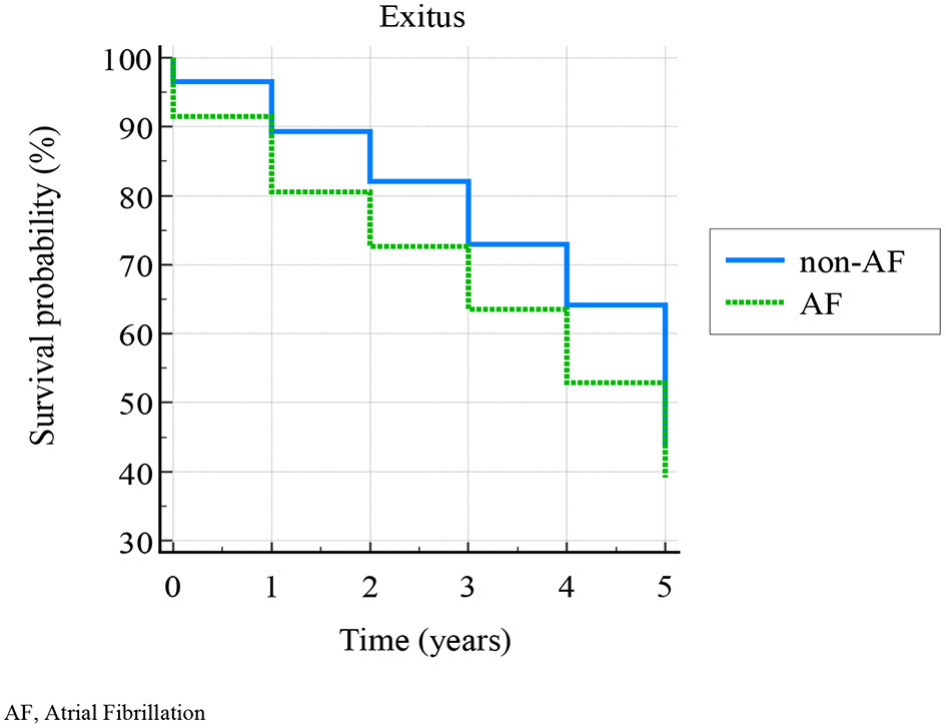
Kaplan-Meier (survive in AF and non-AF group). AF, atrial fibrillation.

Following the aims of the study, we analyzed the data resulting from 647 patients' (98%) follow-up (missing data in 1 patient of AF-group, and 12 patients of non-AF group). The Kaplan-Meier curves (Figure 2) showed a cumulative 58.6% 5-years exitus. According to logrank test, survival probability—calculated at 0, 1, 2, 3, 4, and 5 years—was significantly higher in non-AF than in AF group (χ^2^ = 4.4278, *p* = 0.0354) (Table 2). Non-AF group showed HR: 1.27 (95% CI: 1.01–1.58) for survival.

**Table 2:** Survival proportions.

**Survival time (years)**	**Non-AF group (n. 318)**		**AF group (n. 329)**	
	**Survival probability**	**Standard error**	**Survival probability**	**Standard error**
< 1	0.965	0.0102	0.915	0.0154
1	0.893	0.0173	0.805	0.0218
2	0.821	0.0215	0.726	0.0246
3	0.73	0.0249	0.635	0.0265
4	0.642	0.0269	0.529	0.0275
5	0.437	0.0278	0.391	0.0269

AF, Atrial Fibrillation.

In order to explain the clinical significance of what above reported, we divided CCI scores in three groups (mild severity of comorbidities, scores: 1–2; moderate, scores: 3–4; severe, scores: ≥5), and FRAIL scores in three groups (non-frail, scores: 0; pre-frail, scores: 1–2; frail: ≥3).

**Figure 3:**
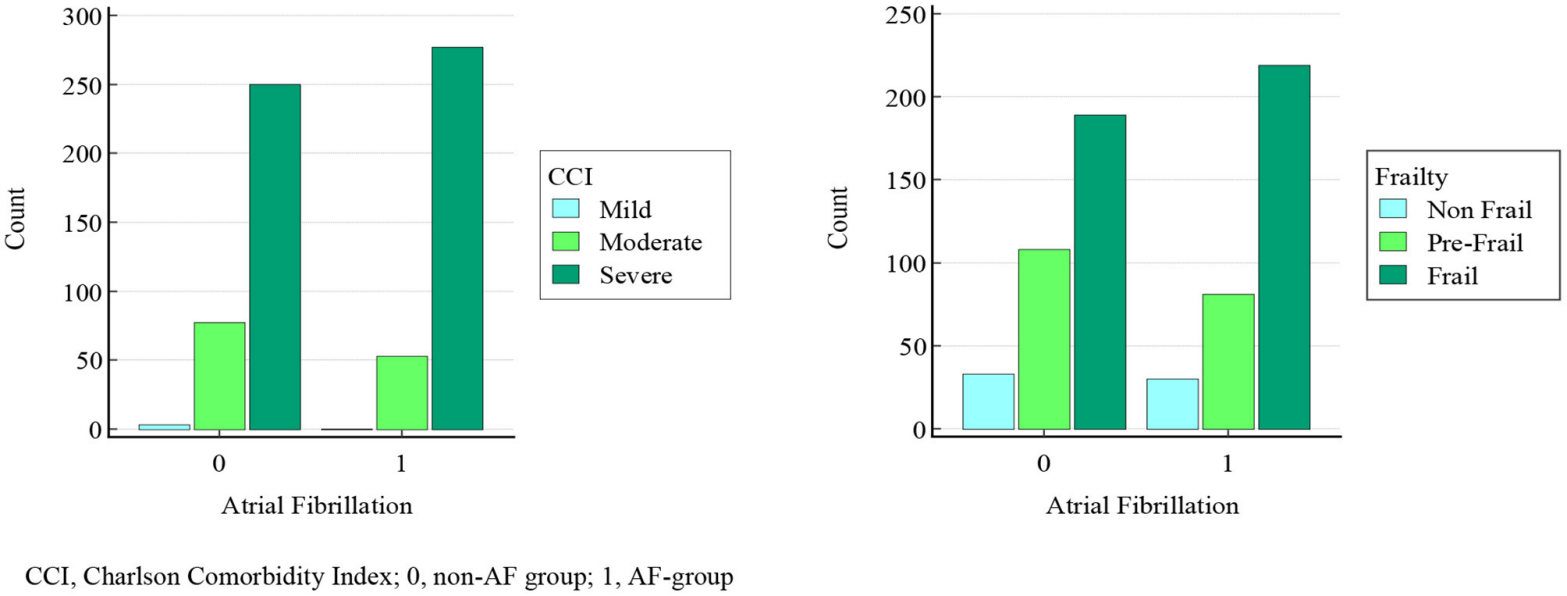
Comparison of comorbidity burden and frailty status in AF and non-AF group. CCI, Charlson Comorbidity Index; 0, non-AF group; 1, AF-group.

As in Figure 3, a moderate severity of comorbidities was found in 53 AF-patients, and 77 non-AF-patients, and severe in 277 AF-patients and 250 non-AF-patients (χ^2^: 8.814, *p* = 0.0122); eighty-one AF-patients and 108 non-AF-patients were pre-frail, and 219 AF-patients and 189 non-AF-patients were frail (χ^2^: 6.206, *p* = 0.0449). Moreover, CCI and FRAIL scores were mutually weakly correlated (*r* = 0.31, *p* < 0.0001), and very weakly correlated with AF (CCI-AF *r* = 0.108, *p* = 0.0005; FRAIL-AF *r* = 0.088, *p* = 0.0231). The collinearity among the other variables (CGA domains, co-morbidities, drugs taken) was also assessed, the large part of which was not significant, and, among the significant ones, only one showed *r* > 0.8 (PPT-POMA, *r* = 0.813, *p* < 0.0001) and two > 0.7 (ADL-PPT, *r* = 0.73, *p* < 0.0001; ADL-POMA, *r* = 0.725, *p* < 0.0001).

AF was then considered as dependent variable in a multivariate logistic regression; CGA domains (cognitive status, mood, autonomy, physical performances, nutritional status, comorbidity burden, and frailty), co-morbidities and drugs taken were considered independent variables (Table 3). The Area Under the ROC Curve (AUC) was 0.808, with a standard error of 0.0203 and a 95% C.I. from 0.769 to 0.844. The regression model demonstrated that the presence of AF was independently associated with a history of coronary heart disease (OR: 2.12, C.I.: 1.13–3.96) and cerebrovascular disease (OR: 1.64, C.I.: 1.01–2.67), with the assumption of Beta Blockers (OR: 3.39, C.I.: 2.09–5.52), and antiplatelets (OR: 0.09, C.I.: 0.05–0.15), and with the amount of drugs taken (OR: 1.12, C.I.: 1.05–1.19).

**Table 3:** Logistic regression – stepwise (y = presence of AF).

Variable^*^	**Coefficient**	**Standard error**	**Odds ratio**	**95% C.I**.	***p***
Coronary heart disease	0.75	0.32	2.12	1.13–3.96	0.0184
Cerebrovascular disease	0.49	0.25	1.64	1.01–2.67	0.0452
Antiplatelet	−2.39	0.27	0.09	0.05–0.15	< 0.0001
Beta blocker	1.22	0.25	3.39	2.09–5.52	< 0.0001
(Total) drugs taken	0.11	0.03	1.12	1.05–1.19	0.0006

* *p* > 0.01 excluded by the model.

C.I., Confidence Interval.

## Discussion

The increasing elderly population is often frail and multimorbid (10), and CGA (6) can early recognize and categorize such common conditions. Among age-related pathologies, one of the most represented is AF, associated with higher risk of hospitalization and mortality (23, 24).

The primary aim of our study was to compare the frailty status and the comorbidity burden with the presence/absence of AF in a population of subjects aged 65 years or older. The secondary aim was to consider which domains, comorbidities and drugs were independently associated with AF.

Our data demonstrated that severe comorbidity burden (*p* = 0.01) and frailty status (*p* = 0.04) were significantly more common in patients with AF than without AF, although their poor collinearity, and such difference did not depend on gender and age, according to the case-control matching performed in our sample. Moreover, non-AF patients were more likely to survive (HR: 1.27) than AFs. These results are consistent with the literature (33–36), and show AF being a disease of serious impact on global health status in elderly patients. In our sample, overall mortality was higher compared to the literature (37), in accordance with our inclusion criteria, and the ensuing abovementioned burden.

Then, we performed a multivariate analysis to characterize the weight of different co-variates on AF. We did not include anticoagulant drugs assumption because of the obvious association with AF, as can be also seen by χ^2^ analysis (*p* < 0.0001). The regression model showed an independent association with coronary and cerebrovascular diseases (ORs: 2.12 and 1.64, respectively), consistently with the literature and with AF's pathophysiology, likewise to Beta Blockers intake (OR: 3.39). A data so far never emerged in the scientific literature (38, 39) was the inverse association between AF and antiplatelets: our data showed that patients without AF have 91% more chance of taking such drugs. To deepen this result, we must consider that, in AF group, 34% of the patients with a history of coronary or cerebrovascular disease did not take any antiplatelet drugs, while only 9.5% of non-AF group did not take them. Moreover, 10% of AF group and 23.5% of non-AF group was taking antiplatelets in primary prevention (40). We can thus highlight the possible tendency to overprescribe (41, 42) such drugs in primary prevention slightly more in absence than in presence of AF, and, on the other hand, the reduced tendency to prescribe them in presence of AF, because of the known increased risk of bleeding (43, 44).

Our study demonstrates that elderly people with AF are frailer, have more severe comorbidities, and take more drugs, in particular beta blockers, and besides have a higher probability to die. Furthermore, it denounces a dangerous antiplatelets underprescription, that might be carefully considered in such population with additional thrombotic risk factors (45).

Obviously, we recognize the study presents some limitation: firstly, its design did not allow to explore causality among the outcomes and the independent variables; moreover, it is monocentric, and it did not take into account potential geographical differences on FA management; lastly, it did not consider the causes of death, which would have been useful in order to enrich the strength of the results.

## Data availability statement

The raw data supporting the conclusions of this article will be made available by the authors, without undue reservation.

## Ethics statement

The studies involving human participants were reviewed and approved by Institutional Review Board (or Ethics Committee) of the University of Cagliari. The patients/participants provided their written informed consent to participate in this study.

## Author contributions

FS, AP, and AM were principal investigators and contributed to the study design and data analyses. FS, AP, GD, and MS contributed to data collection. FS and AM contributed to the interpretation of the findings and wrote the manuscript. All authors read and approved the final version of the manuscript.
